# A new measure of population structure using multiple single nucleotide polymorphisms and its relationship with F_ST_

**DOI:** 10.1186/1756-0500-2-21

**Published:** 2009-02-06

**Authors:** Hongyan Xu, Bayazid Sarkar, Varghese George

**Affiliations:** 1 Deparment of Biostatistics, Medical College of Georgia, Augusta, GA, USA

## Abstract

**Background:**

Large-scale genome-wide association studies are promising for unraveling the genetic basis of complex diseases. Population structure is a potential problem, the effects of which on genetic association studies are controversial. The first step to systematically quantify the effects of population structure is to choose an appropriate measure of population structure for human data. The commonly used measure is *Wright's *F_ST_. For a set of subpopulations it is generally assumed to be one value of F_ST_. However, the estimates could be different for distinct loci. Since population structure is a concept at the population level, a measure of population structure that utilized the information across loci would be desirable.

**Findings:**

In this study we propose an *adjusted C parameter *according to the sample size from each sub-population. The new measure C is based on the *c parameter *proposed for SNP data, which was assumed to be subpopulation-specific and common for all loci. In this study, we performed extensive simulations of samples with varying levels of population structure to investigate the properties and relationships of both measures. It is found that the two measures generally agree well.

**Conclusion:**

The new measure simultaneously uses the marker information across the genome. It has the advantage of easy interpretation as one measure of population structure and yet can also assess population differentiation.

## Background

Large scale genome-wide association studies are promising in unraveling the genetic basis of complex diseases in humans. There are many such studies currently being carried out. However, the size of the data produces several issues and challenges in analysis and interpretation. One of the potential problems is hidden population structure in the samples. It can cause spurious associations when cases and controls differ in ancestry and is thus a confounding factor. However, the effects of population structure in real large-scale association studies are very controversial. Therefore, a systematic study is needed to quantify the levels of population structure and its effects on genetic association studies.

The first step to quantify the effects of population structure is to choose an appropriate measure of population structure for human data. The commonly used measure is Wright's F_ST _[1]. For a set of subpopulations it is generally assumed to be one value of F_ST_. However, the estimates could be different for distinct loci. It could be a problem if population structure is adjusted with local estimates in genome-wide association studies because it could mask real association and lead to loss of power. With the available of genomic data, we would like a measure utilizing the information across markers. Therefore, we proposed a new measure C for SNP data. The new measure is same for all loci and utilizes information across loci. It is based on the c parameters for the subpopulation that measures the divergence of the subpopulation from the common ancestor [2]. We then performed extensive simulations to investigate the performance the new measure and compared it to the traditional F_ST _statistic.

## Methods

### Simulation model

We simulated samples with population structure using the Balding-Nichols model [2,3]. Specifically, let x_ij _be the number of copies of the chosen SNP variant at locus *i *in population *j*, α_ij _be the corresponding allele frequency at locus i in population j, then the model specifies that

$$\begin{array}{l} \begin{array}{cc} x_{ij}\sim \text{Bin}\left(n_{ij},\alpha_{ij}\right) & i=1,\ldots ,L\;j=1,\ldots ,P \end{array}\\ \alpha_{ij}\sim \text{Beta}\left(\frac{\pi_{i}\left(1-c_{j}\right)}{c_{j}},\frac{\left(1-\pi_{i}\right)\left(1-c_{j}\right)}{c_{j}}\right), \end{array}$$

where L is the number of loci, P is the number of populations, *n*_*ij *_is the number of chromosomes genotyped at the *i*th SNP in the *j*th population, π_i _is the ancestral allele frequency for the *i*th SNP and the variance parameter *c*_*j *_specifies how far the *j*th subpopulation's allele frequencies tend to be away from the ancestral allele frequency. In our simulations, we sample *c*_*j *_and π_i _from a uniform distribution on (0, 1). The simulations were performed using the simMD program in the Popgen package [4].

### Estimating c_j_ parameter

For each sample in the simulated data sets, we estimate the *c *parameter for each subpopulation using a Bayesian approach. We assume uniform priors on both *c *and π parameters and use Markov Chain Monte Carlo (MCMC) methods (a Gibbs sampler) to sample from the posterior distribution. The Markov Chain was run for 20,000 iterations and the first 10,000 iterations were discarded as burn-in. We estimated the c parameter by using the posterior mean values from the posterior samples.

### New measure

When summarizing the level of population structure across subpopulations, it is desirable to have a single statistic, instead of one for each subpopulation. We propose to use the weighted mean of the c parameters as the new measure of population structure. Specifically, suppose we have P subpopulations, and let *c*_*j *_be the variance parameter for the *j*th subpopulation as defined above, then the new measure C is defined as



where w_j _is the weight for the *j*th subpopulation. There are many possible weighting schemes. Here we propose to use sample size from each subpopulation as a weight. That is, wj=nj∑i=1Pni, where n_j _is the number of individuals from subpopulation *j *in our sample. In our implementation, we used the posterior estimate of *c*_*j *_and took the weighted mean as an estimate of C.

From the simulated samples, F_ST _was estimated using the unbiased estimator at bi-allelic SNP described by [5].

## Results

### Equal sample size

**Figure 1:**
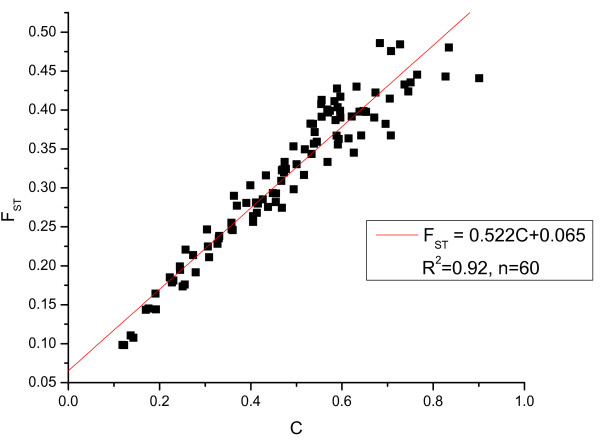
**Linear regression of F_ST _on the estimates of the C parameters with sample size 60 from each of the 3 populations**.

In the first set of simulations, we sampled equal number of chromosomes from each of the 3 sub-populations. We generated genotypes of 100 SNPs according to our simulation model. The posterior estimates of the variance parameter c_j _were obtained using MCMC method. We then estimate C by taking simple average of the estimates of the c_j _parameters. F_ST _was also estimated from the same simulated sample. A total of 100 samples were simulated. The correlation coefficient between the estimates of C and F_ST _was calculated from a linear regression of F_ST _by C. Figure 1 shows the regression of F_ST _by C when the sample size is 60 in each of the 3 sub-populations. As seen in Figure 1, the correlation between the estimates of F_ST _and C is very high (R^2 ^= 0.92). The standard error of the intercept is 0.06.

We then conducted further sets of simulations with varying sample sizes for each set, but equal sample size from each sub-population within each set of simulations. Similar analysis was performed to estimate F_ST_, C, and their correlations. Table 1 gives the correlation coefficient between the estimates of F_ST _and C with varying sample size for several simulations. The number in the sample size column is the number of individuals simulated from each sub-population. Clearly, the correlation between F_ST _and the new measure C increases as the sample size increases. The correlation is high at all the sample sizes we have tried. Even when the sample size is as small as 30 individuals from each sub-population, we still have R^2 ^= 0.89.

**Table 1:** Correlation coefficient of estimates of F_ST _and C from simulations with varying sample sizes

Sample size	Correlation coefficient
30	0.944
60	0.959
90	0.973

To investigate the correlation of C and F_ST _when there are deviations of the Balding-Nichols model, we simulated samples with α_ij _from a mixture of two Beta distributions, with probability (1-a) and a respectively from the two distributions. Table 2 gives the result with a series of values of a. The correlation remains high with 20% mixture.

**Table 2:** Correlation coefficient of estimates of F_ST _and C from simulations with α_ij _from a mixture distribution

Sample size	Correlation coefficient			
	
	a = 0.05	A = 0.10	a = 0.20	a = 0.30
30	0.942	0.925	0.882	0.792
60	0.951	0.939	0.894	0.862
90	0.963	0.946	0.903	0.881

### Unequal sample size

Next we simulated samples with unequal number of individuals from each sub-population. We simulated two scenarios. Specifically, in scenario 1, the number of individuals from the 3 populations are 30, 40, 90, respectively and 30, 60, 70, respectively in scenario 2. The total sample sizes in the two scenarios are the same. For each scenario, we simulated 100 samples, each with information from 100 SNPs. Here because we have unequal sample sizes, we compared 3 weighting schemes for the new measure C. In scheme 1, we estimated C by taking simple average of the c_j _parameters. In scheme 2, we calculated weighted average of c_j _using number of individuals from each sub-population as the weight. In scheme 3, we calculated weighted average of c_j _using the square root of the number of individuals from each sub-population as the weight. Table 3 gives the correlation coefficients from the regression. It can be seen from Table 3 that when sample size is unequal from each sub-population, the simple average in scheme 1 gives the lowest correlation between the estimates of F_ST _and C, while the sample size weighted average in scheme 2 gives the highest correlation. The square root of sample size weighted average in scheme 3 gives a slightly lower correlation than weighting scheme 2. Based on these results, we proposed to use sample size from each sup-population as a weight to estimate the new measure C as detailed in the methods section.

**Table 3:** Correlation of the estimates of F_ST _and C from several weighing schemes

Weighting scheme	Correlation coefficient	
	
	Sample size (30, 40, 90)	Sample size (30, 60, 70)
Scheme 1	0.866	0.875

Scheme 2	0.940	0.947

Scheme 3	0.931	0.937

### Varying number of SNPs

**Figure 2:**
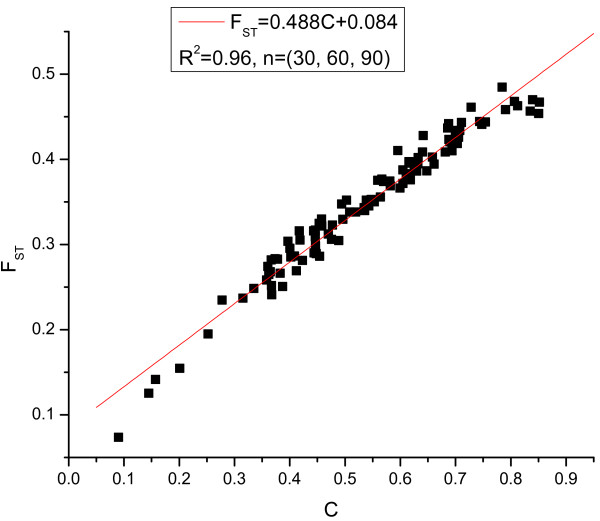
**Linear regression of F_ST _on the estimates of the C parameters with 1000 SNPs**.

Next we consider the effects of the number of SNPs on the correlation between the estimates of F_ST _and the new measure C. In the simulations of previous sections, we simulated samples with 100 SNPs. In this section, we increased the number of SNPs to 1000. We simulated 30, 60, 90 individuals from the three subpopulations respectively. A total of 100 samples were simulated. Figure 2 shows the regression of F_ST _by C with 1000 SNPs. The R^2 ^in this case reaches 0.96. Compared with the case of 100 SNPs and the same sample size configuration (30, 60, 70 from the 3 sub-populations) shown in Table 3, the correlation increases as the number of SNPs increases (R^2 ^= 0.91 in the case of 100 SNPs).

### Varying number of sub-populations

In this section, we studied the effects of varying number of sub-populations on the correlations of the new measure C with F_ST_. We performed further simulations in 3 scenarios with equal sample size from each subpopulation. In scenario 1, we simulated 2 sub-populations with 90 individuals from each sub-population. In scenario 2, 45 individuals were simulated from each of 4 sub-populations. In scenario 3, 36 individuals were simulated from each of 5 sub-populations. Thus, in these simulations, we changed the number of sub-populations but keep the total sample size constant at 180. For each individual, genotypes at 100 SNPs were simulated. We simulated 100 samples. Table 4 gives the correlation coefficient in the 3 scenarios and the simulation with 60 individuals from each of the 3 sub-populations. It can be seen that the correlation increases with more sub-populations even though the total sample size is constant. However, the correlation levels off with 3 or more sub-populations.

**Table 4:** Correlation of the estimates of F_ST _and C with varying number of sub-populations

Sample size	Correlation coefficient
(90, 90)	0.874

(60, 60, 60)	0.959

(45, 45, 45, 45)	0.965

(36, 36, 36, 36, 36)	0.971

## Discussion

Natural populations of the same species from different geographic regions tend to differ genetically. Human population is no exception. Previous research has shown that ignoring the genetic differences among sub-populations is a potential problem for genetic association studies of human diseases, especially for genome-wide association studies [6]. The problem could be severe for large multi-centered studies and/or studies in admixed populations, such as African Americans.

The explosion of SNP data in human populations provides an unprecedented opportunity to further characterize population structure and relationships. In this paper, we proposed a new measure of population structure specifically for SNPs. It is based on the c parameter which is population specific and measures the differentiation of the population from the common ancestor population. In contrast, the new measure C is an index of the overall levels of population structure across populations. Through extensive simulations, we showed that the new measure C has very high correlations with the traditional Wright's F_ST_. The correlation increases as we have more information (more SNPs and/or more sub-populations in the samples).

While the new measure is different from the c parameter, it has some inherited advantages from the c parameters. First, it is specific for SNPs and takes account of the ascertainment bias in the process of SNP discovery. Since SNP discovery is generally conducted in small samples, SNPs with high minor allele frequencies are more likely to be discovered than SNPs with low minor allele frequencies, thus creating the possible ascertainment bias. It has been shown that the ascertainment bias could affect the estimation of population parameters in genetic analysis [7]. This ascertainment bias has been explicitly accounted for in the model for estimating the individual c parameter for each sub-population. It is assumed that a large number of potential loci are examined in small samples from each of the sub-populations, and a locus is chosen if it is not fixed for the same allele in all sub-populations.

Second, the new measure is based on inferences from a Bayesian framework. Therefore, it is very flexible in modeling and can incorporate prior information on the parameters. In our simulation studies, we used uninformative prior distributions for the c and π parameters. If we have any prior knowledge regarding the distribution, we could easily incorporate it in the estimations, which can lead to more accurate estimates than the moment-based estimates of F_ST _[2].

In summary, we proposed a new measure of population structure based on a Bayesian hierarchical model for SNPs. It uses the information at multiple markers and has high correlations with the traditional measure F_ST_. We recommend reporting the new measure along with the individual c parameters for sub-populations so that we could get an idea of the level of population structure and the divergence of each sub-population as well.

## Competing interests

The authors declare that they have no competing interests.

## Authors' contributions

HX conceived of the study, performed the analysis and drafted the manuscript. BS participated in the analysis and helped to draft the manuscript. VG participated in the design and coordination of the study. All authors read and approved the final manuscript.

## References

[B1] Wright S (1943). Isolation by Distance. Genetics.

[B2] Nicholson G, Smith AV, Jónsson F, Gústafsson Ó, Stefánsson K, Donnelly P (2002). Assessing population differentiation and isolation from single-nucleotide polymorphism data. J R Stat Soc Ser B Stat Methodol.

[B3] Balding DJ, Nichols RA (1995). A method for quantifying differentiation between populations at multi-allelic loci and its implications for investigating identity and paternity. Genetica.

[B4] Marchini's Hompage. http://www.stats.ox.ac.uk/~marchini/software.html.

[B5] Weir BS, Cockerham CC (1984). Estimating F-statistics for the analysis of population structure. Evolution.

[B6] Xu H, Shete S (2005). Effects of population structure on genetic association studies. BMC Genet.

[B7] Wakeley J, Nielsen R, Liu-Cordero SN, Ardlie K (2001). The discovery of single-nucleotide polymorphisms – and inferences about human demographic history. Am J Hum Genet.

